# Challenges management in penile calciphylaxis

**DOI:** 10.1016/j.eucr.2022.102219

**Published:** 2022-09-12

**Authors:** Valeerat Swatesutipun, Thoetphum Benyakorn

**Affiliations:** a Division of Urology, Department of Surgery, Faculty of Medicine, Thammasat University Hospital, Thammasat University, Pathum Thani, Thailand; b Division of Vascular Surgery, Department of Surgery, Faculty of Medicine, Thammasat University Hospital, Thammasat University, Pathum Thani, Thailand

**Keywords:** Calciphylaxis, Penile calciphylaxis, Penile ischemia, Arterialization, Revascularization, ESRD, End stage renal disease, CKD, Chronic kidney disease, CABG, Coronary bypass graft, CTA, Computed tomography angiography, PTH, Parathyroid hormone, DUS, Duplex ultrasounography, GSV, Great saphenous vein

## Abstract

Calciphylaxis is a rare, life-threatening vascular disease, which predominantly affects patients with chronic renal failure treated by dialysis. Penile calciphylaxis is an extremely rare condition, a severe manifestation of calciphylaxis, which is associated with poor prognosis and high mortality rate. Diagnosis and management are challenging and still debatable. We present a case with penile calciphylaxis on whom an arterial bypass to the deep dorsal penile vein was performed. Although, in this case, the method was not permanently successful, the histology showed a cluster of neovascularization after the operation. Deep dorsal arterialization might be a proper technique in well-selected patients.

## 1 Introduction

Calciphylaxis is a rare, life-threatening vascular disease which predominantly affect patients with end stage renal disease (ESRD) treated by dialysis. Penile calciphylaxis is a rare condition that is a severe manifestation associated with poor prognosis and a high mortality rate. Diagnosis and management of penile calciphylaxis are challenging and there is no standard of care. Various methods have been attempted to preserve the penis, including arterialization.

## 2 Case presentation

A 46-year-old male with ESRD, diabetes, hypertension, ischemic stroke, and ischemic heart disease presented with penile pain. He had been undergoing hemodialysis since 2016. In 2020, a coronary artery bypass graft (CABG) was performed. After his CABG, he changed the type of dialysis to peritoneal dialysis because of chest pain while undergoing hemodialysis. One year later, he had severe penile pain with a pain score of 9/10, especially at the glans penis. He also reported more intense pain in both hands and the glans penis at cold temperatures.

**Fig. 1:**
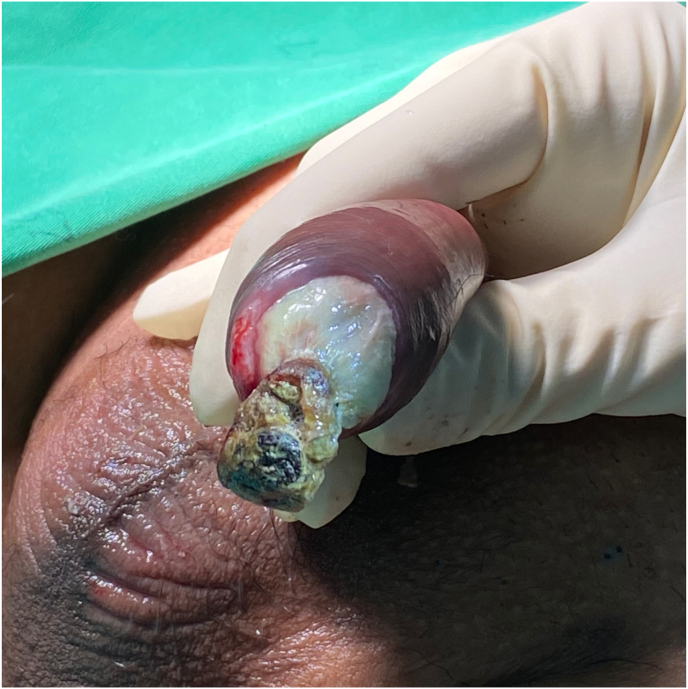
Penile ischemia and gangrene.

**Fig. 2:**
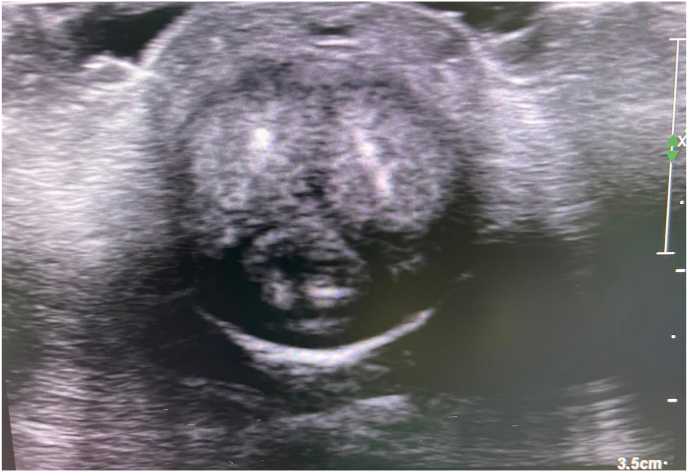
Penile duplex ultrasound demonstrated heavily calcification bilateral cavernosal penile arteries.

**Fig. 3:**
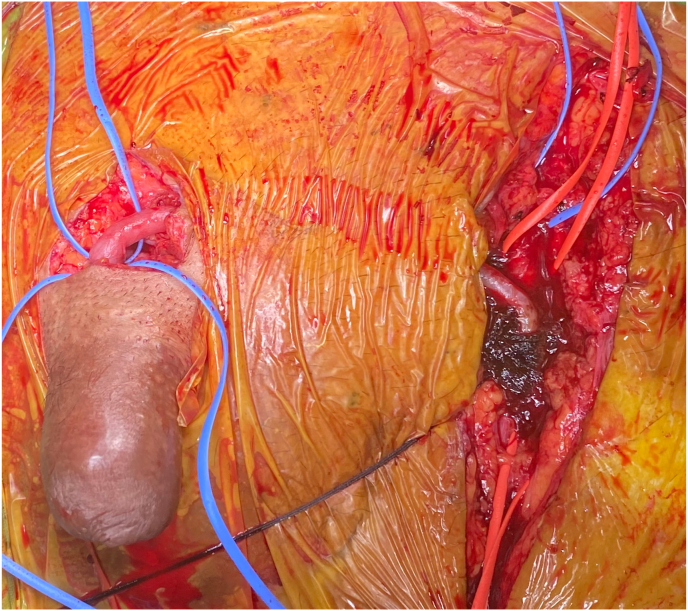
Penile revascularization from left common femoral artery to dorsal penile vein by reverse great saphenous vein graft.

Physical examination revealed mild meatal stenosis and normal color of the glans penis which was treated by dilation. Meanwhile, he developed fingertip ischemic ulcers in both hands. Duplex ultrasonography (DUS) demonstrated sluggish arterial flow in both hands, treated with cilostazol. Two weeks later, the glans of his penis developed pallor and a small area of dry gangrene (Fig. 1). He was treated with unfractionated heparin and sildenafil. The CTA demonstrated heavy vascular calcification. Laboratory investigation revealed albumin 1.71 g/dL, corrected calcium 10.03 mg/dL, phosphorus 6.2 mg/dL, parathyroid hormone (PTH) level 1450.2 pg/mL. Penile DUS showed calcification of cavernosal penile arteries bilaterally with the dramatically decreased flow (Fig. 2). He was diagnosed with calciphylaxis and was treated with sodium thiosulfate, but it was not successful. Then bilateral reverse venous bypass was performed on both hands, and left common femoral artery bypass was performed on the dorsal penile vein, by using reverse great saphenous vein (GSV) concomitant with parathyroidectomy (Fig. 3).

Post-operative pain scores for both hands and penis decreased to 2/10, and the penile and finger necrosis did not progress. Two months after the procedure, he developed severe penile pain with sepsis. Physical examination revealed progressive penile necrosis with pus discharge from the left corpus cavernosum. Total penile amputation with suprapubic cystostomy was performed. Intraoperative findings included infected pseudoaneurysm of GSV graft, wet necrosis at entire left corpus cavernosum, and normal corpus spongiosum.

The pathology report indicated suppurative inflammation and focal necrosis of bilateral cavernous cavernosa and pseudoaneurysm of the deep dorsal vein (DDV), arteriosclerosis of the right dorsal artery up to 40% luminal stenosis, calciphylaxis of the small arteries with near-complete (>90%) to focally complete luminal stenosis. It is interesting to note that the pathological examination also found focal recanalization, which was marked in the bilateral cavernosal arteries, bilateral urethral arteries, and left dorsal artery and neovascularization. After amputation, his wound healed well without complication.

Six months later, the patient died from acute myocardial infarction.

## 3 Discussion

Calciphylaxis is associated with significant morbidity and mortality. The one-year mortality rate is about 45–80%; sepsis is the most common cause of death.^1^ The pathophysiology of calciphylaxis is calcification, fibrointimal hyperplasia, and thrombosis of the microvessels in the subcutaneous adipose tissue and dermis, which often progress to necrosis. Risk factors of calciphylaxis are obesity, diabetes mellitus, female sex, hypercalcemia, hyperphosphatemia, hyperparathyroidism, hypoalbuminemia, duration of dialysis of more than 2 years.^2^ Common manifestation of this disease is painful skin lesion which can progress to necrosis.

Penile calciphylaxis is a rare condition that is a severe manifestation associated with poor prognosis and high mortality rate.^3^ Most patients who have penile involvement are aged between 40 and 60 years. Diagnosis is challenging at the early phase because patients have only penile pain without skin lesion; therefore, physicians should be more suspicious of this disease in patients with ESRD presenting with painful penis. The high level of calcium, phosphorus and PTH is helpful in diagnosis, but it might be normal in some patients.^2^ Biopsy should be avoided because it could increase risk of infection and lead to wet gangrene. Doppler ultrasound can aid in the assessment of the patency of vessel lumen and penile blood flow.

The treatment of penile calciphylaxis is still controversial. The management strategies are local wound care, pain control, and preserving the blood supply to the penis. It can start with conservative therapy, in cases that have not developed necrosis yet. Sodium thiosulfate can be used to delay the development of further calcification. If the conservative approach fails, most patients end up with partial or total penectomy. A novel treatment for penile calciphylaxis is hyperbaric oxygen therapy.^4^

Femoral artery to dorsal penile vein bypass was first proposed by Tu et al.^5^ They performed deep dorsal vein arterialization in an ESRD patient with penile ischemia. The dorsal penile artery and corpora cavernosal artery had severe calcification and atherosclerosis, therefore these vessels were unsuitable for anastomosis. DDV arterialization increased blood supply to the cavernous body and improved blood supply to the surrounding skin.^5^ DDV arterialization has been used in some patients with vasculogenic impotence.

In our case, the patient wanted to preserve his penis; therefore, we performed partial penectomy of the gangrene glans and DDV arterialization by using a reversed GSV graft. We exposed the deep dorsal penile vein at the root of the penis. Proximal and distal anastomoses were side-to-side anastomoses, and the GSV was subcutaneously tunneled. After the operation, the patient's pain was immediately relieved, and expansion of the necrotic lesion stopped. Although he developed further necrosis and infection 2 months later, the pathological report showed neovascularization, which was an effect of arterialization. No standard management has proven to be successful in treating severe cases. Although our outcome was imperfect, this procedure might preserve the penis in well-selected and non-severe patients.

## 4 Conclusion

Penile calciphylaxis is an extremely rare occurrence with a high mortality rate. Although, arterial to vein bypass failed in our patient, the histology showed a neovascular cluster in the penile tissue. This procedure may pave the way for treating in well selected and non-severe patients.

## Funding

None.

## Financial disclosure

None.

## Ethical consideration

This case report was approved by The Human Research Ethics Committee of Thammasat University (Medicine), MTU-EC-SU-0-142/64. The Human Research Ethics Committee of Thammasat University (Medicine) is in full compliance with international guidelines such as Declaration of Helsinki, The Belmont Report, CIOMS Guidelines and the International Conference on Harmonisation-Good Clinical Practice (ICH-GCP). This case report was informed and consented by the patient.

## Author contributions

Valeerat Swatesutipun; Conceptualization, Data curation, Investigation, Methodology, Project administration, Resources, Validation, Visualization, Roles/Writing - original draft, Writing - review & editing.

Thoetphum Benyakorn; Conceptualization, Data curation, Investigation, Methodology, Project administration, Resources, Validation, Visualization, Roles/Writing - original draft, Writing - review & editing.

## Ethics approval

Ethical approval to report this case was obtained from * The Human Research Ethics Committee of Thammasat University (Medicine) (APPROVAL NUMBER: MTU-EC-SU-0-142/64)*.

## Informed consent

Written informed consent was obtained from the patient(s) for their anonymized information to be published in this article.

## Declaration of competing interest

The Authors declare that there is no conflict of interest.
